# OculusNet: Detection of retinal diseases using a tailored web-deployed neural network and saliency maps for explainable AI

**DOI:** 10.3389/fmed.2025.1596726

**Published:** 2025-07-02

**Authors:** Muhammad Umair, Jawad Ahmad, Oumaima Saidani, Mohammed S. Alshehri, Alanoud Al Mazroa, Muhammad Hanif, Rahmat Ullah, Muhammad Shahbaz Khan

**Affiliations:** ^1^Faculty of Engineering, Multimedia University, Cyberjaya, Malaysia; ^2^Cybersecurity Center, Prince Mohammad Bin Fahd University, Al Khobar, Saudi Arabia; ^3^Department of Information Systems, College of Computer and Information Sciences, Princess Nourah bint Abdulrahman University, Riyadh, Saudi Arabia; ^4^Department of Computer Science, College of Computer and Information Sciences, Najran University, Najran, Saudi Arabia; ^5^Department of Informatics, School of Business, Örebro Universitet, Örebro, Sweden; ^6^School of Computer Science and Electronic Engineering (CSEE), University of Essex, Colchester, United Kingdom; ^7^School of Computing, Engineering and the Built Environment, Edinburgh Napier University, Edinburgh, United Kingdom

**Keywords:** retina, retinal disorder, explainable AI, artificial intelligence, ophthalmic imaging, neural networks

## Abstract

Retinal diseases are among the leading causes of blindness worldwide, requiring early detection for effective treatment. Manual interpretation of ophthalmic imaging, such as optical coherence tomography (OCT), is traditionally time-consuming, prone to inconsistencies, and requires specialized expertise in ophthalmology. This study introduces OculusNet, an efficient and explainable deep learning (DL) approach for detecting retinal diseases using OCT images. The proposed method is specifically tailored for complex medical image patterns in OCTs to identify retinal disorders, such as choroidal neovascularization (CNV), diabetic macular edema (DME), and age-related macular degeneration characterized by drusen. The model benefits from Saliency Map visualization, an Explainable AI (XAI) technique, to interpret and explain how it reaches conclusions when identifying retinal disorders. Furthermore, the proposed model is deployed on a web page, allowing users to upload retinal OCT images and receive instant detection results. This deployment demonstrates significant potential for integration into ophthalmic departments, enhancing diagnostic accuracy and efficiency. In addition, to ensure an equitable comparison, a transfer learning approach has been applied to four pre-trained models: VGG19, MobileNetV2, VGG16, and DenseNet-121. Extensive evaluation reveals that the proposed OculusNet model achieves a test accuracy of 95.48% and a validation accuracy of 98.59%, outperforming all other models in comparison. Moreover, to assess the proposed model's reliability and generalizability, the Matthews Correlation Coefficient and Cohen's Kappa Coefficient have been computed, validating that the model can be applied in practical clinical settings to unseen data.

## 1 Introduction

Retinal imaging technologies, such as optical coherence tomography (OCT), have become essential tools in ophthalmology due to their high resolution, non-invasive nature, and ability to reveal critical microstructural details of retinal layers. In humans and most vertebrates, the retina is a thin, light-sensitive membrane located at the back of the eye (1). It consists of several layers, including one made up of light-sensitive cells known as photoreceptors. The retina converts incoming light into neural signals (2–4). The human eye contains two types of photoreceptors: rods and cones. Rod photoreceptors are responsible for black-and-white vision and motion detection, performing particularly well in low-light conditions. Cone photoreceptors, on the other hand, are responsible for color and central vision. These receptors function well in medium to bright light. Rods occupy the entire retina; however, cones are located and clustered in a small central area of the retina known as the macula. Furthermore, there is a slight depression at the center of the macula, called the fovea. The fovea is the point in the retina primarily responsible for color vision and visual acuity (the sharpness of eyesight). The captured information is processed by the retina and transmitted to the brain through the optic nerve for further visual recognition (2, 4, 5). All of these parts of the retina are critical for eyesight, and most eyesight-related diseases primarily occur due to damage or disease in the retina (3, 6).

Several diseases can damage the retina, including choroidal neovascularization (CNV), diabetic macular edema (DME), and age-related macular degeneration characterized by drusen. These disorders lead to visual impairment and even blindness. Conditions affecting the retina have a critical impact on patients, as eyesight (i.e., vision) plays a vital role in human life. Therefore, scientists have been exploring new and effective tools for diagnosing and detecting retinal conditions early (2, 7, 8). Recently, OCT has proven to be a promising non-invasive technique for micro-scale imaging of biological tissues (9). OCT technology captures images of the retina in a cross-sectional format using light waves (10, 11). OCT is significant in various medical applications, with ophthalmology being its largest commercial application. OCT is preferred for the monitoring and detection of several retinal diseases (12, 13). This technology has evolved through various configurations since its inception, i.e., time-domain OCT (14), spectral-domain OCT (15), and swept-source OCT (16). Owing to the aforementioned technological advancements, OCT is the most preferred and reliable method for diagnosing eye diseases.

Recently, the field of biomedicine has significantly evolved in the detection and analysis of diseases. Traditional methods of disease detection were often unreliable and time-consuming. However, with the advent of artificial intelligence (AI), accuracy in disease detection has increased exponentially, and turnaround times have greatly improved. Deep learning (DL) techniques have become integral to the biomedical field, providing fast and reliable results for disease detection. Therefore, this paper presents an efficient and explainable approach to classify retinal disorders, such as CNV, DME, and Drusen, from normal conditions.

The main contributions presented by this study include the following:

1. An efficient and explainable DL model specifically designed for complex medical image patterns in OCTs classifies retinal disorders, such as CNV, DME, and Drusen, differentiating them from normal conditions. The results of reliability parameters validate that OculusNet can be effectively used in practical settings on unseen data.
2. The proposed approach utilizes saliency map visualization, an explainable AI (XAI) technique, to visualize the most influential pixels and interpret how the proposed model makes its decisions while identifying retinal disorders. Results from Saliency Maps have also been compared to other XAI techniques, such as GradCam++, SHAP, and Lime.
3. The trained weights of the OculusNet model are deployed on a webpage using the Streamlit server, which is accessible to all devices connected to the same network. This deployment demonstrates significant potential for integration into ophthalmic departments, enhancing diagnostic accuracy and efficiency.
4. The transfer learning technique has been applied to ensure a fair comparison. It has been applied to four state-of-the-art models that have been proven to be the best for classification problems. The utilized pre-trained models include VGG16, VGG19, MobileNetV2, and DenseNet-121.

The structure of the rest of the article is organized as follows: Section 2 presents the literature related to this study, Section 3 discusses the dataset and data pre-processing methodologies, Section 4 presents the utilized methodology along with the design and architecture of the proposed OculusNet model, and Section 5 discusses the performance parameters that are utilized for this study and reports the obtained results. Finally, a conclusion to this study is provided in Section 6.

## 2 Related research

OCTs provide high-resolution cross-sectional images of the retina, which play a potential role in diagnosing retinal disorders (17). Recently, DL has been extensively utilized to detect retinal disorders efficiently (11, 18–20). The development of novel image processing models has enhanced noise reduction and retinal layer segmentation in OCT images, thereby facilitating the accurate diagnosis of retinal disorders (21). The application of OCT in pediatric ophthalmology has been revolutionary, enabling the visualization of retinal structures in infants and neonates (6), which is crucial for the diagnosis of retinal-related diseases. The integration of deep learning (DL) methods has addressed reliability issues in OCT image analysis, leading to improved diagnostic accuracy (18, 22). Additionally, OCT's capability to differentiate between retinoschisis and retinal detachment has been confirmed, showcasing its diagnostic versatility. For the diagnosis of age-related macular degeneration diseases, several methods have been proposed that detect retinal pigment epithelium (RPE) through OCT images (23). Recently, various algorithms have also been developed for the classification of eye diseases. For example, the authors in Muni Nagamani and Rayachoti (24) present a DL approach that utilizes OCT images for the classification of retinal diseases using a modified ResNet50 model. Their study shows that they used a single-view retinal image set along with applied segmentation. Similarly, in Wang et al. (25), a semi-supervision-based approach named Caps-cGAN has been proposed to reduce noise in OCT images, particularly speckle noise. Furthermore, to extend the automated diagnosis of eye diseases, a semi-automated approach is presented in Shin et al. (26). The authors utilized pig eye images in this approach and achieved an accuracy of 83.89%. The classification of retinal diseases, namely DME, Drusen, and CNV, has been performed in Adel et al. (27), using the Inception and Xception models with 6,000 OCT images.

Recent literature also demonstrates the use of pre-trained models for detecting retinal disorders. For instance, in Islam et al. (28), a total of 109,309 images were utilized for four classes: CNV, Drusen, DME, and Normal, using 11 pre-trained models. Similarly, several pre-trained models have been presented to demonstrate the effectiveness of CNNs in detecting various retinal diseases (29–31).

Moreover, the authors in Jawad et al. (32) apply four Swin Transformer variants to multi-classify fundus images, utilizing local window self-attention to address the limited global modeling of traditional CNNs. Swin-L outperforms earlier research, with final scores reaching up to 0.97 and an AUC exceeding 0.95 on three ODIR test splits and an external retina dataset, demonstrating strong generalization. Metric standard deviations remain below 0.05, and one-way ANOVA indicates non-significant differences among models (*p* = 0.32–0.94), confirming the statistical stability of their results.

Furthermore, in a study by Abdul Jawad and Khursheed (33), the authors build a DenseNet-based deep-and-dense CNN that classifies BreakHis breast-cancer slides into benign/malignant subtypes across all magnifications, achieving up to 96.6% patient-level and 91.8% image-level accuracy, with *t*-tests showing that the gains over earlier CNNs are significant. Additionally, in another study by Abdul Jawad and Khursheed (34), the authors introduce an automatic, color-balanced reference-image selector that, when paired with Reinhard, Macenko, and Vahadane normalization, consistently boosts SSIM, QSSIM, and PCC on BreakHis and BACH datasets; Wilcoxon tests confirm that the improvements compared to random selection are also significant.

Furthermore, the authors in Bhandari et al. (35) proposed a lightweight convolutional neural network with only 983,716 trainable parameters. They employed this architecture to classify OCT images of three retinal pathologies: CNV, DME, and Drusen, achieving a test accuracy of 94.29% and a validation accuracy of 94.12%. To interpret the model's decisions, the authors applied two explainable AI techniques: Local Interpretable Model-Agnostic Explanations (LIME) and Shapley Additive exPlanations (SHAP), which highlighted clinically relevant retinal regions. The same network was subsequently tested on two additional medical imaging tasks: COVID-19 detection from chest X-rays and kidney stone classification. Similarly, in another study by Bhandari et al. (41), the authors utilized the proposed model with explainable AI techniques, including LIME, Gradient-weighted Class Activation Mapping (Grad-CAM), and SHAP.

Thus, the majority of existing approaches lack interpretability and explanations of the decision-making process, which is extremely important in clinical settings. Therefore, in addition to designing a tailored deep learning model for the efficient and accurate detection of retinal disorders, this paper focuses on integrating explainable AI to visualize and interpret the features on which the identification of retinal disorders is based.

## 3 Data preparation

### 3.1 Dataset

**Figure 1:**
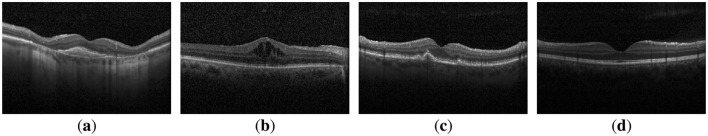
Samples of the dataset images of each class. **(a)** CNV-disorder class. **(b)** DME-disorder class. **(c)** Drusen-disorder class. **(d)** Normal-no disorder class.

In this study, retinal OCT images were used to train DL models for the classification of retinal disorders. From the accessed dataset [“Retinal OCT Images (optical coherence tomography)” data (36)], a balanced, high-quality subset of 6,200 images (1,550 per class) was constructed using a two-step procedure: quality screening and class balancing with computational constraints. During quality screening, many raw files were found to contain white borders or artifacts unrelated to retinal tissue. These were excluded to prevent the models from learning irrelevant or misleading patterns. For class balancing with computational limits, all experiments were conducted on Google Colab, where limited GPU memory could not accommodate the entire dataset without frequent crashes. A balanced subset of 1,550 images per class provided a practical compromise between dataset representativeness and hardware constraints. The dataset comprises four classes: normal, CNV disorder, DME disorder, and Drusen disorder. It was divided into three sets: training, testing, and validation. A two-stage split strategy was used. In the first stage, the dataset was split into 80% for training and 20% for testing. In the second stage, the training set was further divided, with 80% used for model training and 20% for validation. Details of the dataset are provided in Table 1, and representative sample images of retinal OCT scans are shown in Figure 1.

**Table 1:** Summary of dataset classes and distributions.

**Class name**	**Splitting details**			
	**Training set**	**Validation set**	**Testing set**	**Total**
CNV	992	248	310	1,550
DME	992	248	310	1,550
Drusen	992	248	310	1,550
NORMAL	992	248	310	1,550

### 3.2 Data preprocessing and augmentation

**Figure 2:**
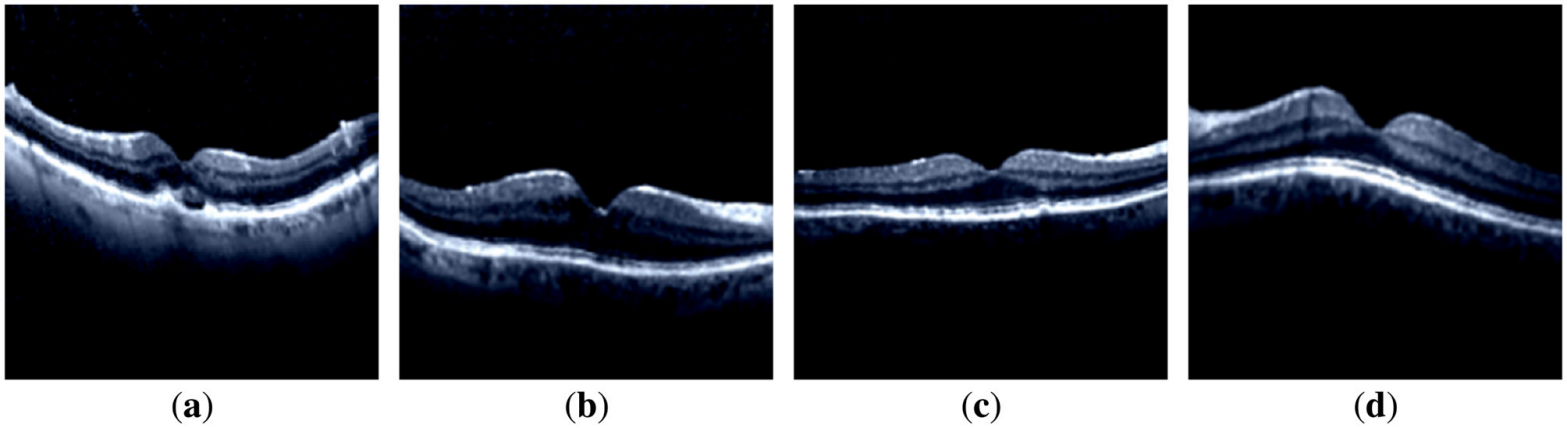
Images after preprocessing: **(a)** CNV-disorder class; **(b)** DME-disorder class; **(c)** Drusen-disorder class; and **(d)** Normal-no disorder class.

To train the DL on the OCT images, the dataset was preprocessed. The OCT images in the dataset primarily contain noisy pixels. Noisy pixels lead to incorrect feature extraction during model training, resulting in underfitting and overfitting issues. To eliminate noisy pixels, a preprocessing function *Prep*_*func*_ given in Equation 1 was utilized, which normalizes image pixels from the range [0, 255] to [−1, 1]. Samples of the preprocessed and rescaled images for each model are displayed in Figure 2. It is evident that there are no noisy pixels (compared to the sample images shown in Figure 1).


(1) $$\begin{array}{c} Prep_{func}=\left(\left(\frac{image\;pixels}{255}\right)-0.5\right)\times 2 \end{array}$$

**Figure 3:**
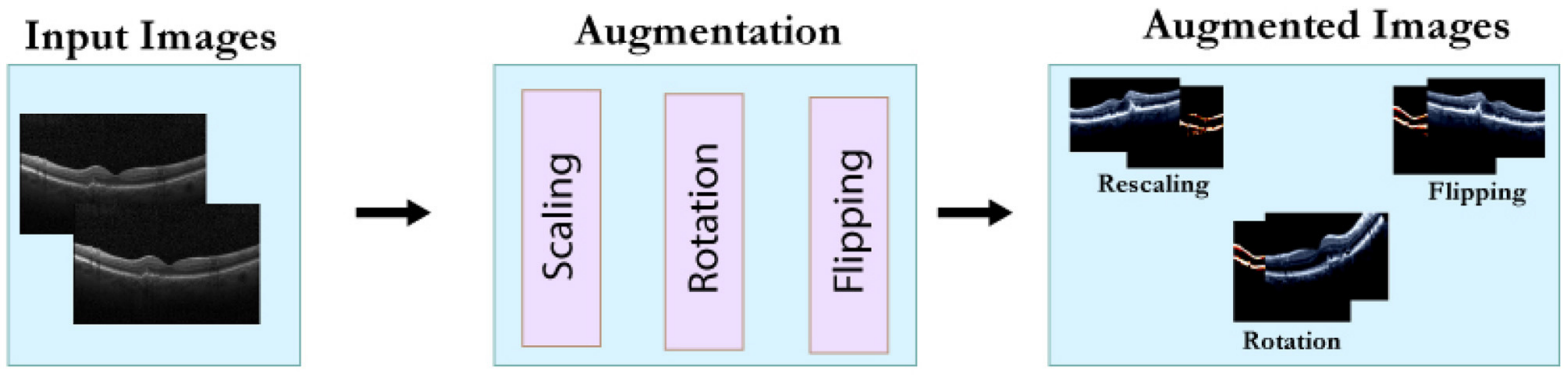
Data augmentation process.

After scaling the images, data augmentation was applied using a 30° rotation and horizontal flipping. Samples of the augmented images are illustrated in Figure 3.

## 4 Methodology and experiments

**Figure 4:**
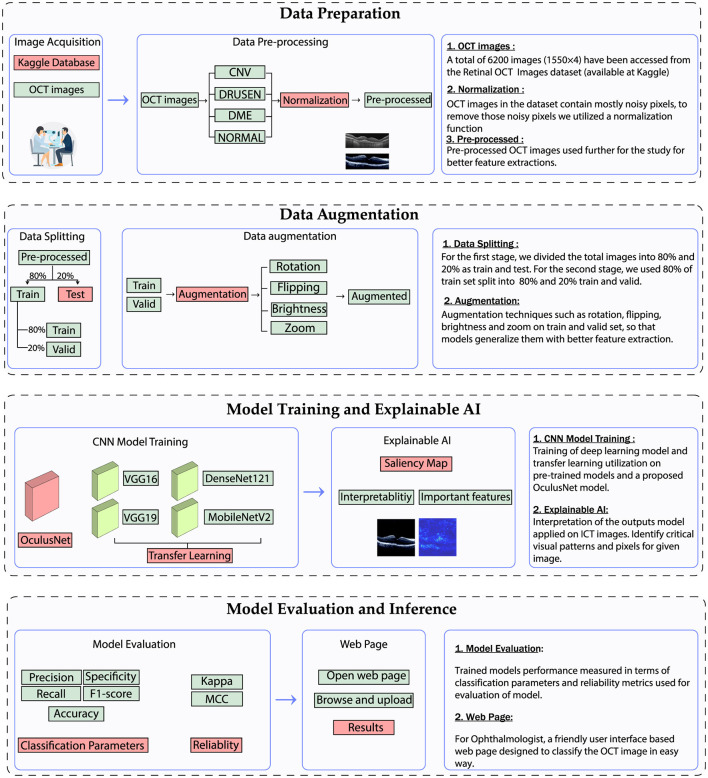
Utilized methodology for the classification of retinal diseases.

The methodology employed in this study consists of four key stages: data preparation, data augmentation, model training with explainable AI, and model evaluation. An overview of the employed methodology is illustrated in Figure 4. The data preparation and data augmentation processes have already been discussed in the previous section. This section details the model architecture and evaluation parameters.

### 4.1 The proposed OculusNet model

#### 4.1.1 Architecture of the proposed model

**Figure 5:**
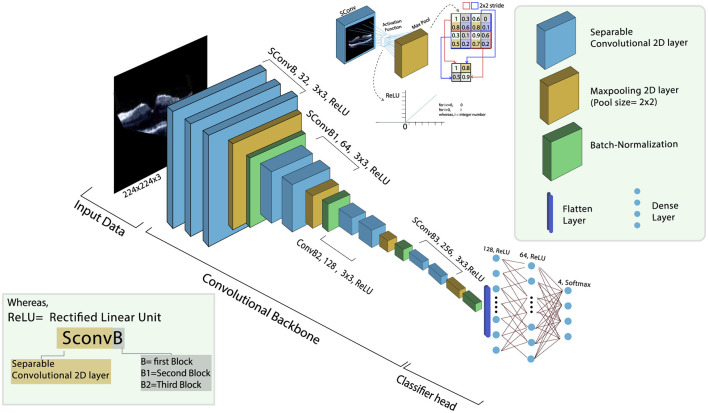
Architectural overview of the proposed OculusNet model.

A DL model named OculusNet has been proposed in this study. The OculusNet model comprises nine depthwise separable (DWS) convolutional layers, four max pooling layers, and four batch normalization layers. A max pool size of 2 × 2 has been kept for this model. For each DWS layer, a kernel size of 3 × 3 has been utilized, and in each 2D separable convolutional layer, the “ReLU” activation function is utilized. The model summary and parameter information for each layer are shown in Table 2. The model architecture of the proposed CNN (i.e., OculusNet) is depicted in Figure 5.

**Table 2:** Summary of layer outputs and parameters.

**Layers**	**Output shape**	**Parameters**
separable_conv2d	222, 222, 32	155
separable_conv2d_1	220, 220, 32	1,344
separable_conv2d_2	218, 218, 32	1,344
Max_Pooling2d	109, 109, 32	0
Batch_Normalization	109, 109, 32	128
separable_conv2d_3	107, 107, 64	2,400
separable_conv2d_4	105, 105, 64	4,763
Max_Pooling2d_1	52, 52, 64	0
Batch_Normalization_1	52, 52, 64	256
separable_conv2d_5	50, 50, 128	8,896
separable_conv2d_6	48, 48, 128	17,664
Max_Pooling2d_2	24, 24, 128	0
Batch_Normalization_2	24, 24, 128	512
separable_conv2d_7	22, 22, 256	34,176
separable_conv2d_8	20, 20, 256	68,096
Max_Pooling2d_3	6, 6, 256	0
Batch_Normalization_3	6, 6, 256	1,024
Flatten	9216	0
Dense	128	1,179,776
Dense_1	64	8,256
Dense_2	4	260

#### 4.1.2 Depthwise separable convolutional layers

**Figure 6:**
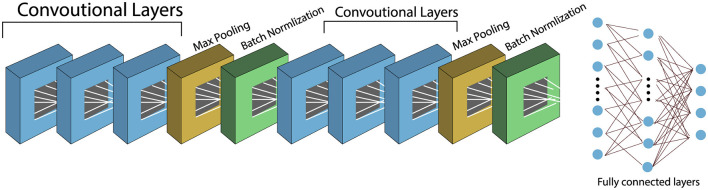
Standard convolutional neural layers.

**Figure 7:**
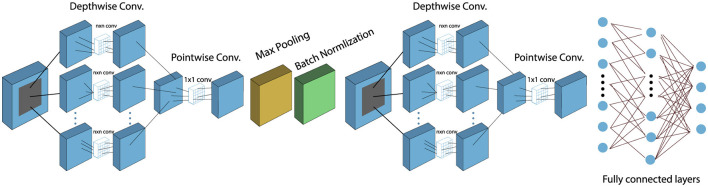
Depthwise convolutional neural layers.

Depthwise Separable Convolution (DWS) is a computationally efficient alternative to standard convolution, designed to reduce the number of trainable parameters and floating-point operations in convolutional neural networks. It decomposes a standard convolution into two distinct operations: *depthwise* convolution and *pointwise* convolution (37, 38). The visual comparison between standard and DWS convolutional layers is illustrated in Figures 6, 7, respectively.

In a standard convolutional layer, an input image tensor with the shape *A*_*h*_×*A*_*w*_×*N* (where *A*_*h*_ and *A*_*w*_ represent the height and width, and *N* signifies the number of input channels) is convolved with *n* filters, each sized *K*_*d*_×*K*_*d*_×*N*. This produces an output tensor of shape $A_{h}^{\prime }\times A_{w}^{\prime }\times n$, where $A_{h}^{\prime }$ and $A_{w}^{\prime }$ depend on the stride and padding. The computational cost for this operation is described by Equation 2:


(2) $$\begin{array}{c} S=A_{h}\times A_{w}\times K_{d}^{2}\times N\times n \end{array}$$

In contrast, depthwise separable convolution divides the operation described above into two stages:

1. **Depthwise convolution:** Applies a single *K*_*d*_×*K*_*d*_ filter to each input channel (no cross-channel mixing), generating *N* feature maps. The computational cost is as follows:

(3) $$\begin{array}{c} DW=A_{h}\times A_{w}\times K_{d}^{2}\times N \end{array}$$

2. **Pointwise convolution:** Applies 1 × 1 × *N* filters to combine the output of the depthwise stage across channels, generating *n* output channels. The computational cost is as follows:

(4) $$\begin{array}{c} PW=A_{h}\times A_{w}\times N\times n \end{array}$$

The total cost of a depthwise separable convolution is the sum of Equations 3, 4, as indicated in Equation 5:


(5) $$\begin{array}{c} CM=DW+PW=A_{h}\times A_{w}\times \left(K_{d}^{2}\times N+N\times n\right) \end{array}$$

To understand the efficiency of DWS, the ratio of its computational cost to that of standard convolution (Equation 2) is derived in Equation 6:


(6) $$\begin{array}{c} \frac{CM}{S}=\frac{K_{d}^{2}\times N+N\times n}{K_{d}^{2}\times N\times n}=\frac{1}{n}+\frac{1}{K_{d}^{2}} \end{array}$$

For instance, with *n* = 256 filters and a kernel size of *K*_*d*_ = 3, Equation 6 yields:


(7) $$\begin{array}{c} \frac{CM}{S}=\frac{1}{256}+\frac{1}{9}\approx 0.115 \end{array}$$

This indicates that depthwise separable convolution requires only about 11.5% of the computational cost of standard convolution while still producing feature representations with comparable effectiveness. This significant reduction in operations and parameters makes DWS particularly suitable for lightweight architectures such as OculusNet, which are intended for deployment in resource-constrained environments such as mobile or web applications.

Figure 6 illustrates the standard convolution operation, where each filter operates on all input channels simultaneously. In contrast, Figure 7 shows the DWS operation, which performs filtering channel-wise followed by channel mixing, effectively decoupling spatial and cross-channel computations.

#### 4.1.3 Workflow of the utilized architecture

The coding flow utilized for the experiments was organized into four main stages: data preprocessing, building model architecture, model training, and model evaluation. Initially, the necessary libraries were imported, followed by the dataset, where preprocessing functions were applied to standardize images and split the dataset into training, validation, and testing subsets. Hyperparameters were selected, and data augmentation techniques were employed using an image data generator library to enhance the model's generalization capability. For building the models, the OculusNet model was defined from scratch by adding convolutional, max-pooling, flatten, and dense layers, after which a model summary was printed. Similarly, pre-trained models were imported from Keras libraries with ImageNet weights, followed by adding flatten and dense layers and printing their summaries. During model training, the models were trained on the training dataset and validated on the validation dataset, while loss and accuracy graphs were generated. The model evaluation step involved obtaining the confusion matrices and classification reports for both validation and testing datasets to comprehensively assess the models' performance. This structured approach ensures a coherent and efficient workflow, leading to reliable and reproducible results.

### 4.2 Explainable AI using saliency maps

DL models are typically referred to as black box models due to the complicated interpretation of outputs or predicted results from the trained DL models. However, several visualization techniques are available, such as Grad-CAM (39–41), and saliency maps, which are often known as class activation maps. By using saliency maps, one can compute the effect of each input pixel on the final prediction, highlighting the influential pixels in the image that the model uses to classify the given image. However, Grad-CAM does not calculate pixel by pixel; instead, it generates a heatmap of the input pixels (39, 40).

In this study, saliency maps have been utilized for class-specific results on images. Mathematically, the saliency map can be explained by Equation 8, where *w* is the weight of each pixel. *S*_*n*_ represents the score of the specific class *n*, which is acquired by the trained model. *I* represents the pixel's values of the given image. Additionally, other techniques, such as Grad-CAM, have also been employed (Grad-CAM++ is selected for this case), along with LIME and SHAP.


(8) $$\begin{array}{c} w=\frac{6S_{n}}{6I} \end{array}$$

### 4.3 Transfer learning on pre-trained models for comparison

**Figure 8:**
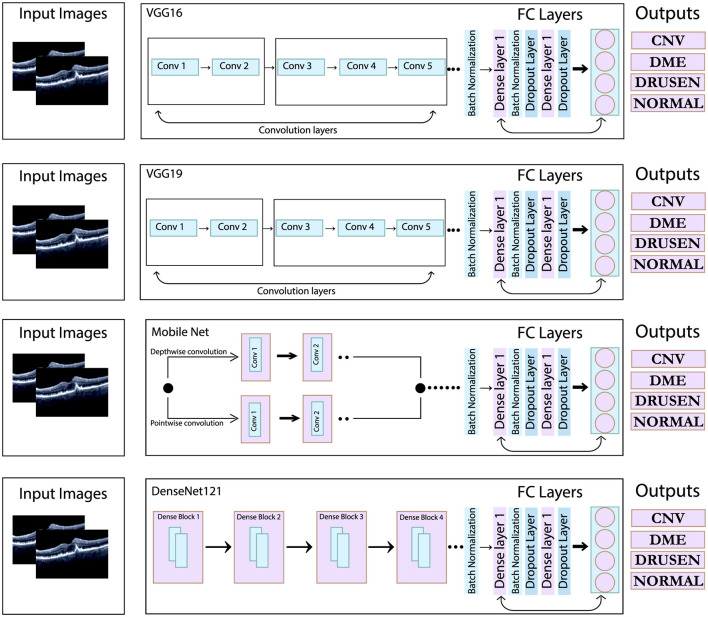
Architectural overview of the pre-trained model used in this study.

To ensure a fair comparison, transfer learning has been employed to optimize pre-trained models that are well-known for classification tasks. The models used include VGG-16, VGG-19, MobileNetV2, and DenseNet-121. The VGG16 model consists of 13 convolutional layers and three dense layers, while the VGG19 model comprises 16 convolutional layers with three dense layers. In contrast, MobileNetV2 is a lightweight neural network with fewer parameters, comprising 28 convolutional layers that utilize depthwise separable convolution. Lastly, the DenseNet121 model includes 121 layers organized into four dense blocks. The architectural details of the aforementioned models are shown in Figure 8.

The aforementioned models are pre-trained on ImageNet, a large dataset comprising 1,000 classes and almost 1,281,167 images. In this study, the convolutional layers of the aforementioned models were frozen to utilize the pre-trained weights, and the fully connected layers were replaced to retrain the model for classifying retinal OCT images. Additionally, to prevent underfitting during the training phase, batch normalization layers and a dropout layer with a size of 0.25 were added to the fully connected layers. The details regarding the layers and parameters of all models are provided in Table 3, which includes the input layer size of 224 × 224 × 3 (*height*×*width*×*dimension*) and an output layer size of 4, 1, where 4 represents the four classes and 1 is the final output layer.

**Table 3:** Model architectures and layer details.

**Model name**	**No. of layers**	**Size of input layer**	**Size of output layer**
VGG19	19	(224, 224, 3)	(4, 1)
VGG16	16	(224, 224, 3)	(4, 1)
MobileNet	28	(224, 224, 3)	(4, 1)
DenseNet-121	121	(224, 224, 3)	(4, 1)
OculusNet	12	(224, 224, 3)	(4, 1)

Moreover, the trainable and non-trainable parameters of the utilized models are detailed in Table 4. These parameters are often referred to as model summaries and are derived from the parameters used in the layers of the model. For the pre-trained models, non-trainable parameters were the sum of the frozen layers (frozen with ImageNet weights), while the trainable parameters were the sum of the FC layer parameters (for which the model weights were not frozen). The weights of the model were updated only for the trainable parameters during the training phase. Additionally, the FC layers remain the same for all the utilized models. The first dense layer uses the ReLU activation function with 128 units, and the second dense layer consists of 64 units. The third dense layer, which is the output layer, has four units and uses softmax as the activation function. Softmax, in this context, is responsible for providing the output.

**Table 4:** Model parameters and size details.

**Model name**	**Total parameters**	**Trainable parameters**	**Non-trainable parameters**	**Model size (MB)**
VGG19	23,246,340	3,220,932	20,025,408	16.54
VGG16	17,936,644	3,220,932	14,715,712	26.21
MobileNet	9,664,132	6,433,220	3,230,912	6.22
DenseNet-121	13,472,772	6,433,220	7,039,552	26.56
OculusNet	1,329,023	1,328,063	960	5.07

## 5 Experiment and results

### 5.1 Evaluation parameters

#### 5.1.1 Performance parameters

For the experimental parameters, a grid search strategy was used, with the number of epochs set to 100 and the batch size set to 32. A learning rate of 0.00001 with RMS optimizer was applied. For performance evaluation, key performance parameters, including precision, recall, specificity, F1 score, and accuracy, were employed. These parameters were derived from the confusion matrices. The 4 × 4 confusion matrix with character representation is given in Table 5. The analysis of the confusion matrix has been presented for both the validation and testing datasets. Considering the characters used in Table 5, the TP and TN samples for each respective class are listed in Table 6. Similarly, the FP and FN samples for each respective class are provided in Table 7.

**Table 5:** Representation of the confusion matrix.

**Confusion matrix**				
	**Predicted classes**			
**Actual classes**	**CNV**	**DME**	**Drusen**	**NORMAL**
**CNV**	TP_*CNV*_	F_*AB*_	F_*AC*_	F_*AD*_
**DME**	F_*BA*_	TP_*DME*_	F_*BC*_	F_*BD*_
**Drusen**	F_*CA*_	F_*CB*_	TP_*Drusen*_	F_*CD*_
**NORMAL**	F_*DA*_	F_*DB*_	F_*DC*_	TP_*NORMAL*_

**Table 6:** Class-wise true positive and true negative.

**Class**	**True positive (TP)**	**True negative (TN)**
**CNV**	TP_*CNV*_	TP_*DME*_ + F_*BC*_ + F_*BD*_ + F_*CB*_ + TP_*Drusen*_ + F_*CD*_ + F_*DB*_ + F_*DC*_ + TP_*NORMAL*_
**DME**	TP_*DME*_	TP_*CNV*_ + F_*AC*_ + F_*AD*_ + F_*CA*_ + TP_*Drusen*_ + F_*CD*_ + F_*DA*_ + F_*DC*_ + TP_*NORMAL*_
**Drusen**	TP_*Drusen*_	TP_*CNV*_ + F_*AB*_ + F_*AD*_ + F_*BA*_ + TP_*DME*_ + F_*BD*_ + F_*DA*_ + F_*DB*_ + TP_*NORMAL*_
**NORMAL**	TP_*NORMAL*_	TP_*CNV*_ + F_*AB*_ + F_*AC*_ + F_*BA*_ + TP_*DME*_ + F_*BC*_ + F_*CA*_ + F_*CB*_ + TP_*Drusen*_

**Table 7:** Class-wise false positives and false negatives.

**Class**	**False positive (FP)**	**False negative (FN)**
**CNV**	F_*BA*_ + F_*CA*_ + F_*DA*_	F_*AB*_ + F_*AC*_ + F_*AD*_
**DME**	F_*AB*_ + F_*CB*_ + F_*DB*_	F_*BA*_ + F_*BC*_ + F_*BD*_
**Drusen**	F_*AC*_ + F_*BC*_ + F_*DC*_	F_*CA*_ + F_*CB*_ + F_*CD*_
**NORMAL**	F_*AD*_ + F_*BD*_ + F_*CD*_	F_*DA*_ + F_*DB*_ + F_*DC*_

**Accuracy** represents the model's overall ability to correctly classify the TP and TN classes from all the predicted labels. It can be calculated using Equation 9.


(9) $$\begin{array}{c} \text{Accuracy}=\frac{TP+TN}{FP+TP+TN+FN}\times 100 \end{array}$$

**Precision** measures the actual TP samples from all the positive predicted samples of the respective class. It can be calculated using Equation 10.


(10) $$\begin{array}{c} \text{Precision}=\frac{TP}{TP+FP} \end{array}$$

**Recall** is also commonly known as sensitivity. It measures the actual TP samples from the considered predicted samples of that class. It can be calculated using Equation 11.


(11) $$\begin{array}{c} \text{Recall}=\frac{TP}{FN+TP} \end{array}$$

**Specificity** measures the TN samples from the predicted samples of the classes. It can be calculated using Equation 12.


(12) $$\begin{array}{c} \text{Specificity}=\frac{TN}{TN+FP} \end{array}$$

**F1-Score** describes the actual predicted results obtained through precision and recall. It can be calculated using Equation 13.


(13) $$\begin{array}{c} F1-\text{score}=2\times \frac{\text{Precision}\;\times \;\text{Recall}}{\text{Precision}\;+\;\text{Recall}} \end{array}$$

#### 5.1.2 Reliability parameters

To assess the model's reliability on unseen data and its generalization, reliability parameters such as Cohen's kappa coefficient and Matthews correlation coefficient have been calculated.

**Cohen's Kappa coefficient**

Cohen's Kappa statistic (kappa) assesses a model's performance by measuring the agreement between predicted and actual labels, while accounting for the agreement that could happen by chance. It indicates how accurate the predictions are, or how close they are to the actual value of their respective labels. It is computed using the observed and expected accuracy values extracted from the confusion matrices. Observed accuracy corresponds to the ratio of accurately predicted values to the total number of values in the confusion matrix. Equation 14 represents the mathematical representation for observed accuracy. Expected accuracy is defined as a random accuracy, and Equation 15 shows the mathematical representation of expected accuracy, where the sum of total values in the predicted row for a respective class is multiplied by the sum of total values of the actual class for the respective class, and *i*∈0, 1, 2, 3 represents the four classes: 0: CNV, 1: DME, 2: Drusen, and 3: NORMAL used in this study. The combination of observed accuracy versus expected accuracy is known as the kappa statistic, and Equation 16 represents the kappa statistic.


(14) $$\begin{array}{c} \text{Observed accuracy}=\frac{TP_{i}+TN_{i}}{TP_{i}+TN_{i}+FP_{i}+FN_{i}} \end{array}$$

(15) $$\begin{array}{l} \text{Expected accuracy}=\\ \frac{\left(TP_{i}+FP_{i}\right)\left(TP_{i}+FN_{i}\right)+\left(TN_{i}+FP_{i}\right)\left(TN_{i}+FN_{i}\right)}{{\left(TP_{i}+TN_{i}+FP_{i}+FN_{i}\right)}^{2}} \end{array}$$

(16) $$\begin{array}{c} \text{Kappa}=\frac{\text{Observed Accuracy}\;-\;\text{Expected Accuracy}}{1\;-\;\text{Expected Accuracy}} \end{array}$$

**Matthews correlation coefficient**

The Matthews correlation coefficient (MCC) is used to assess the correlation between predicted and true binary classification labels, taking into account all four confusion matrix categories (TP, TN, FP, FN). The range of MCC lies between –1 and 1, where 1 indicates that a model is perfectly positive and capable of classifying positive samples with greater accuracy. Conversely, –1 indicates that the model has a negative correlation and, in most cases, will misclassify positive samples. Thus, –1 represents the worst-case scenario for a model, which will be unable to classify the samples correctly. Equation 17 represents the mathematical formulation of MCC, and *i* ∈0, 1, 2, 3 represents the four classes, namely 0: CNV, 1: DME, 2: Drusen, and 3: NORMAL, used in this study.


(17) $$\begin{array}{l} MCC=\frac{\left(TP_{i}\times TN_{i}\right)-\left(FP_{i}\times FN_{i}\right)}{\sqrt{\left(TP_{i}+FP_{i}\right)\left(TP_{i}+FN_{i}\right)\left(TN_{i}+FP_{i}\right)\left(TN_{i}+FN_{i}\right)}}\\ \text{ where, }i\in 0,1,2,3\; \end{array}$$

### 5.2 Results and discussion

This section provides the results of the training performance, the key performance parameters, and the reliability parameters of the models. In addition, an ablation study has also been performed on the proposed OculusNet model to validate it.

#### 5.2.1 Training and validation results

**Figure 9:**
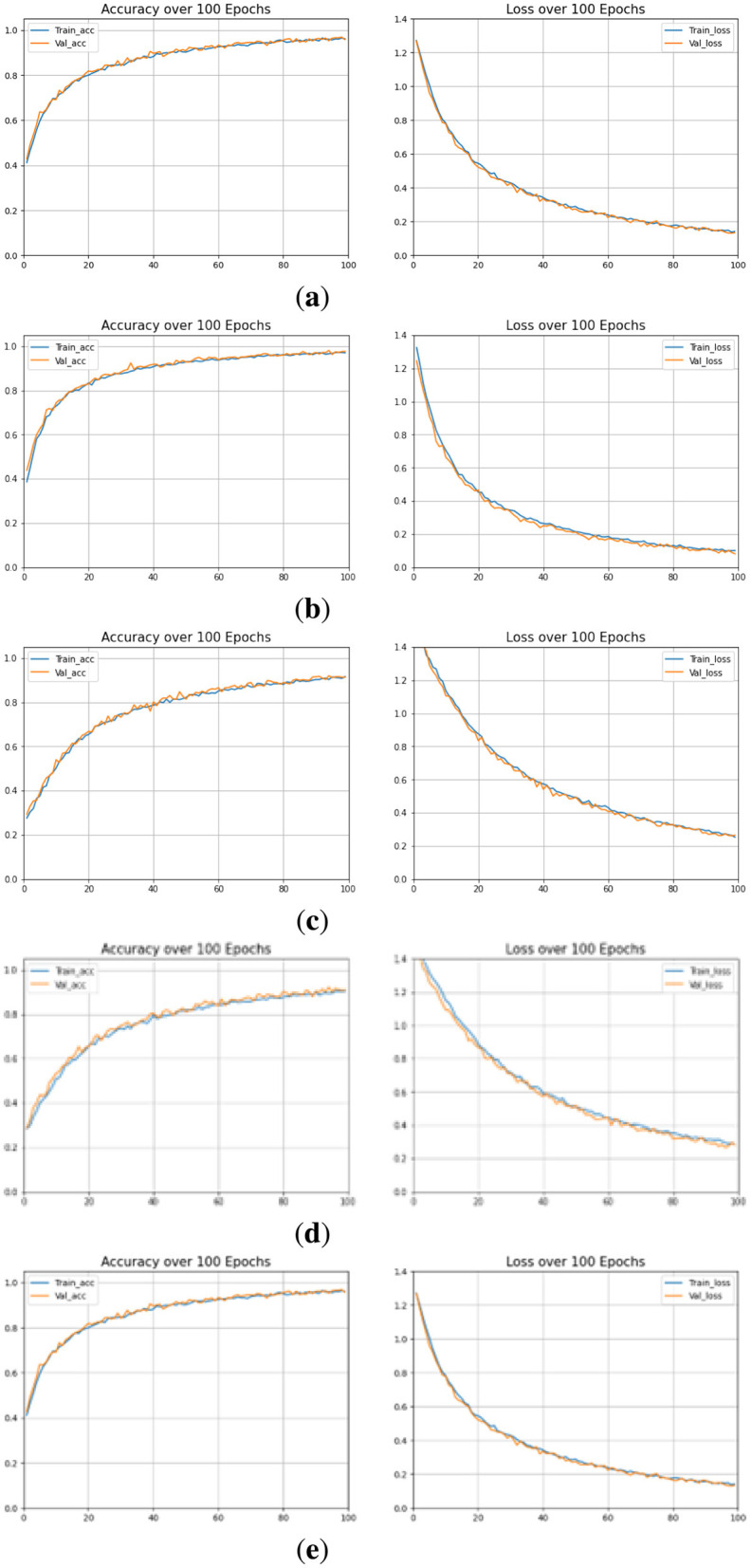
Accuracy and Loss curves. **(a)** DenseNet121. **(b)** MobileNetV2. **(c)** VGG16. **(d)** VGG19. **(e)** OculusNet.

The training performance of the proposed OculusNet model, as well as the models used for comparison, has been evaluated and reported in terms of validation and test accuracy. The training curves for the proposed OculusNet model, along with the other utilized models, are shown in Figure 9. The curves demonstrate that the models are well-trained, exhibiting no data bias, underfitting, or overfitting. Each model achieved over 90% validation and training accuracy.

#### 5.2.2 Class-wise classification report

**Figure 10:**
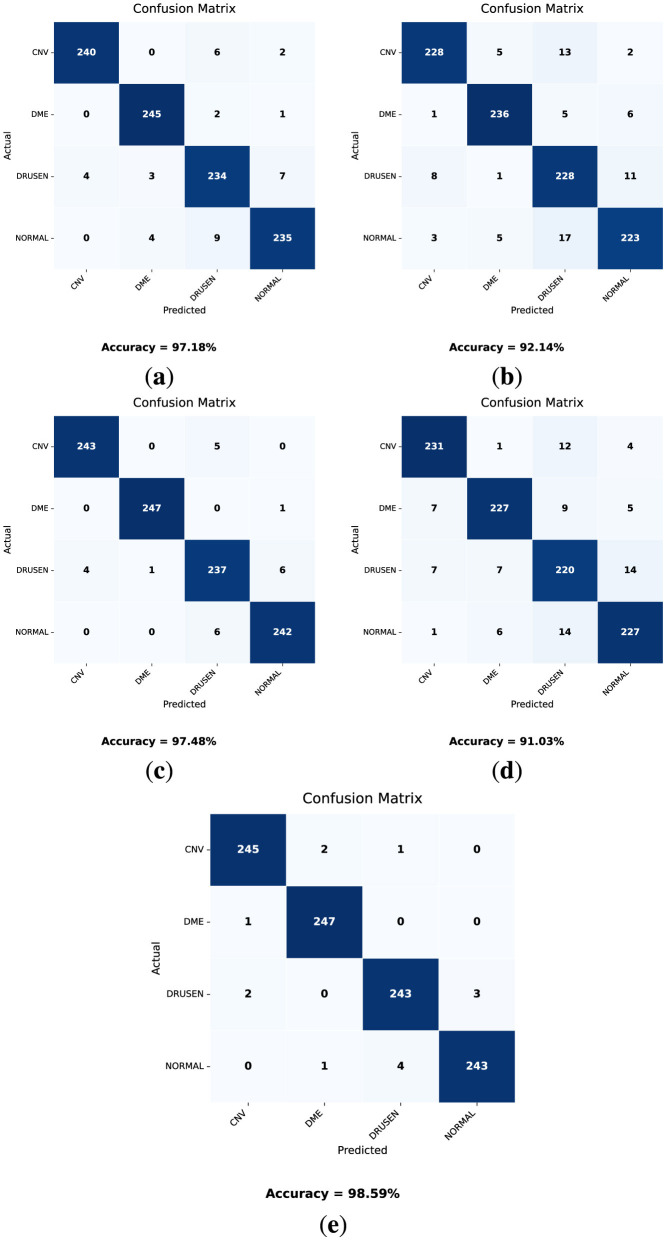
Confusion matrices for the validation dataset for each model. **(a)** DenseNet121. **(b)** VGG16. **(c)** MobileNetV2. **(d)** VGG19. **(e)** OculusNet.

The confusion matrices obtained from the validation dataset are shown in Figure 10. In these confusion matrices, the labels on the y-axis represent the actual labels, while the labels on the x-axis represent the predicted number of images for the respective classes. Moreover, Table 8 provides a classification report for each model. These parameter values are obtained after the training of the model and can be validated using Equations 9–13.

**Table 8:** Performance parameters of the models used for the validation dataset.

**Models**	**Classes**	**Precision**	**Recall**	**F1-Score**	**Specificity**
DenseNet121	CNV	0.983	0.967	0.974	0.994
	DME	0.972	0.987	0.979	0.990
	Drusen	0.932	0.943	0.937	0.979
	Normal	0.959	0.947	0.952	0.986
VGG16	CNV	0.950	0.919	0.934	0.983
	DME	0.955	0.951	0.952	0.985
	Drusen	0.866	0.919	0.891	0.952
	Normal	0.921	0.899	0.909	0.974
MobileNetV2	CNV	0.983	0.979	0.98	0.994
	DME	0.995	0.995	0.995	0.998
	Drusen	0.955	0.955	0.955	0.985
	Normal	0.971	0.975	0.972	0.990
VGG19	CNV	0.939	0.931	0.935	0.980
	DME	0.942	0.915	0.928	0.981
	Drusen	0.863	0.887	0.875	0.953
	Normal	0.908	0.915	0.912	0.969
OculusNet	CNV	0.987	0.987	0.987	0.995
	DME	0.988	0.995	0.991	0.995
	Drusen	0.979	0.979	0.979	0.993
	Normal	0.987	0.979	0.982	0.995

Considering the results presented in Table 8 and the obtained confusion matrices shown in Figure 10, the validation accuracies of each model “VGG19, DenseNet121, MobileNetV2, and VGG16” are 91.03%, 97.18%, 97.48%, and 92.14%, respectively. However, the proposed model, OculusNet, outperformed the pre-trained models, achieving a validation accuracy of 98.59%.

**Figure 11:**
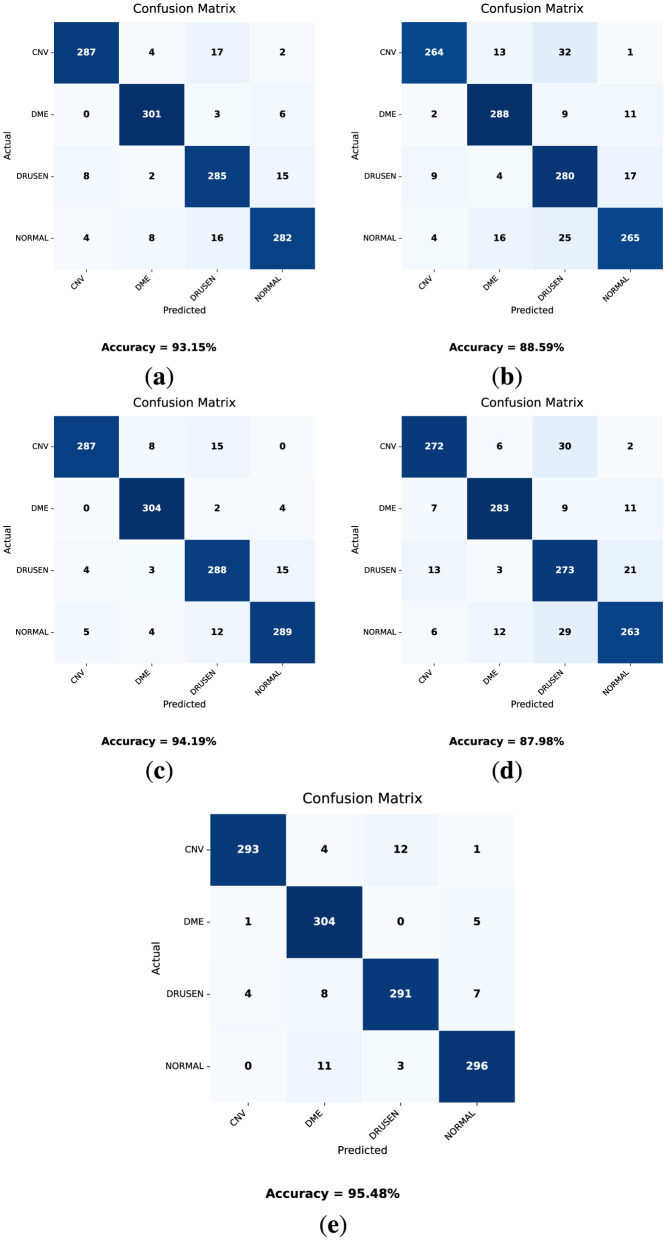
Confusion matrices on the test dataset for each model **(a)** DenseNet121. **(b)** VGG16. **(c)** MobileNetV2. **(d)** VGG19. **(e)** OculusNet.

Similarly, the confusion matrices (Figure 11) and classification report (Table 9) for the testing dataset have been obtained. The test dataset remains unseen and was not exposed to the models during the training phase.

**Table 9:** Performance parameters of the utilized models for the test dataset.

**Models**	**Classes**	**Precision**	**Recall**	**F1-Score**	**Specificity**
DenseNet121	CNV	0.960	0.926	0.943	0.987
	DME	0.956	0.971	0.963	0.985
	Drusen	0.888	0.919	0.903	0.961
	Normal	0.925	0.910	0.917	0.975
VGG16	CNV	0.946	0.852	0.896	0.984
	DME	0.897	0.929	0.913	0.965
	Drusen	0.809	0.903	0.854	0.929
	Normal	0.901	0.855	0.877	0.969
MobileNetV2	CNV	0.969	0.925	0.947	0.990
	DME	0.953	0.980	0.966	0.983
	Drusen	0.908	0.929	0.918	0.968
	Normal	0.938	0.935	0.935	0.979
VGG19	CNV	0.912	0.877	0.894	0.972
	DME	0.930	0.912	0.921	0.974
	Drusen	0.800	0.880	0.838	0.926
	Normal	0.885	0.848	0.866	0.963
OculusNet	CNV	0.983	0.945	0.963	0.994
	DME	0.929	0.980	0.953	0.975
	Drusen	0.950	0.938	0.943	0.983
	Normal	0.957	0.954	0.955	0.986

Considering the results from these models as shown in Table 9 and Figure 11, the test accuracy for each model “VGG19, DenseNet121, MobileNetV2, and VGG16” was 87.98%, 93.15%, 94.19%, and 88.59%, respectively. The proposed model achieved the highest test accuracy of 95.48%. These results indicate that the proposed model outperformed all other pre-trained models with the highest validation and testing accuracies.

#### 5.2.3 Reliability parameters results

The results of the reliability parameters are presented in Table 10, demonstrating that the proposed OculusNet model outperformed across all classes and metrics, with observed accuracies ranging from 0.989 to 0.995 on the validation set and 0.972 to 0.982 on the test set, along with high Kappa and MCC values. This suggests that OculusNet's architecture is particularly effective for feature extraction and generalization capabilities suited to the specific characteristics of retinal disease images. The Kappa statistic and MCC values across all models and classes were high, indicating a strong agreement between the predicted and actual labels. These metrics highlight the models' ability to accurately differentiate among various classes, which is crucial in medical diagnostics, where the stakes are high due to the potential consequences of misdiagnosis. The high Kappa and MCC values also imply that the models were well-trained, providing a reliable assessment of their predictive performance.

**Table 10:** Kappa coefficient and Matthews correlation coefficient for all models.

**Dataset**	**Models**	**Classes**	**Expected Acc**.	**Observed Acc**.	**Kappa**	**MCC**
Valid	DenseNet-121	CNV	0.627	0.987	0.965	0.967
		DME	0.622	0.989	0.970	0.973
		Drusen	0.623	0.968	0.915	0.917
		NORMAL	0.626	0.976	0.935	0.937
	VGG16	CNV	0.629	0.967	0.911	0.913
		DME	0.625	0.976	0.936	0.938
		Drusen	0.617	0.944	0.853	0.855
		NORMAL	0.628	0.955	0.879	0.880
	MobileNetV2	CNV	0.625	0.990	0.973	0.975
		DME	0.625	0.997	0.992	0.994
		Drusen	0.625	0.977	0.938	0.940
		NORMAL	0.624	0.986	0.962	0.965
	VGG19	CNV	0.626	0.967	0.911	0.913
		DME	0.628	0.964	0.903	0.905
		Drusen	0.621	0.936	0.831	0.832
		NORMAL	0.623	0.955	0.880	0.882
	OculusNet	CNV	0.625	0.993	0.981	0.983
		DME	0.623	0.995	0.986	0.989
		Drusen	0.625	0.989	0.970	0.971
		NORMAL	0.626	0.991	0.975	0.978
**Test**	DenseNet-121	CNV	0.629	0.971	0.921	0.924
		DME	0.622	0.981	0.949	0.950
		Drusen	0.620	0.950	0.868	0.870
		NORMAL	0.627	0.958	0.887	0.889
	VGG16	CNV	0.637	0.950	0.862	0.866
		DME	0.620	0.955	0.881	0.883
		Drusen	0.610	0.922	0.800	0.803
		NORMAL	0.631	0.940	0.837	0.838
	MobileNetV2	CNV	0.630	0.974	0.929	0.930
		DME	0.621	0.983	0.955	0.955
		Drusen	0.622	0.958	0.888	0.891
		NORMAL	0.625	0.967	0.912	0.913
	VGG19	CNV	0.629	0.948	0.859	0.860
		DME	0.627	0.961	0.895	0.896
		Drusen	0.612	0.915	0.780	0.783
		NORMAL	0.630	0.934	0.821	0.823
	OculusNet	CNV	0.629	0.982	0.951	0.952
		DME	0.618	0.976	0.937	0.939
		Drusen	0.626	0.972	0.925	0.926
		NORMAL	0.625	0.978	0.941	0.941

#### 5.2.4 Saliency map results

**Figure 12:**
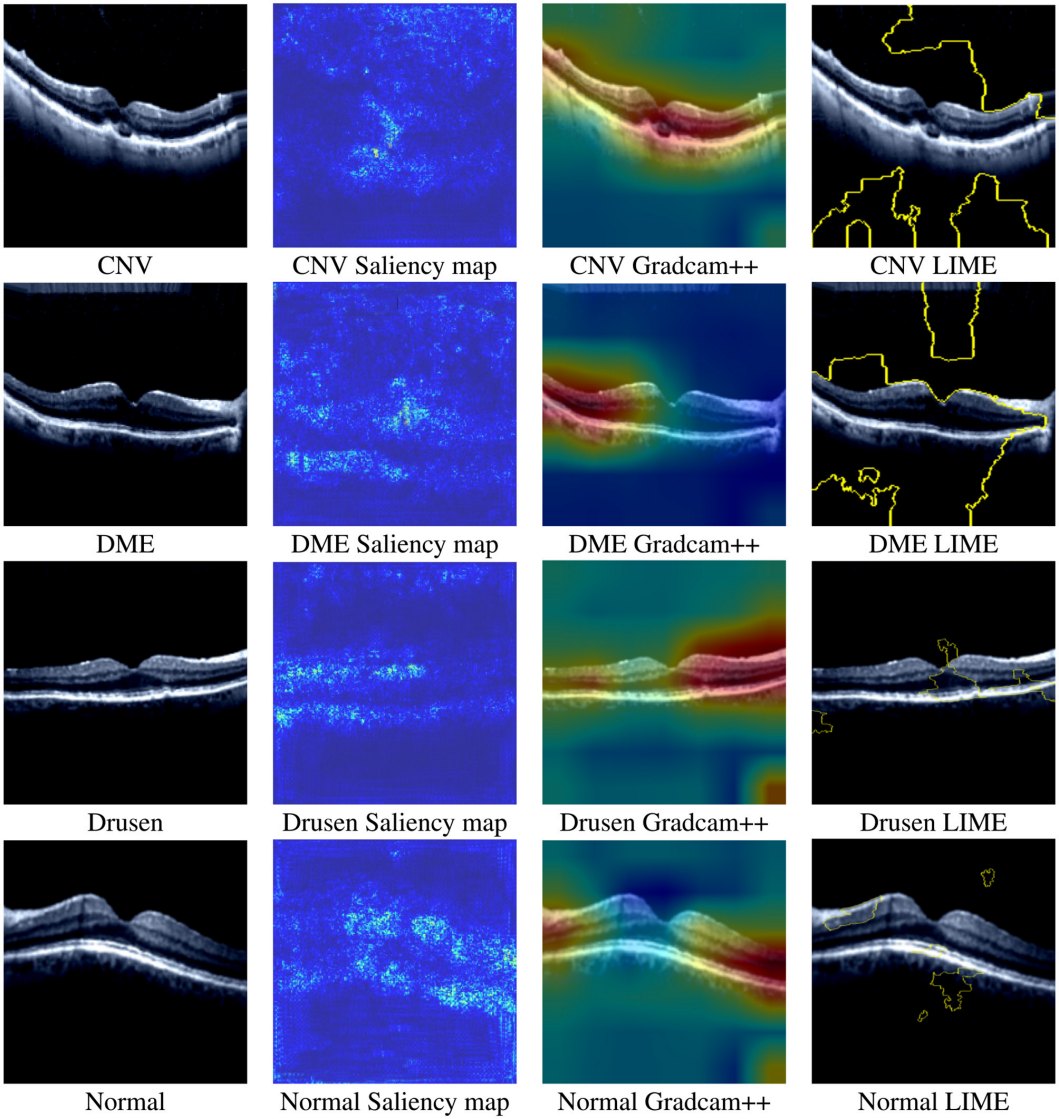
Saliency maps, Gradcam++, and LIME heatmaps generated by the trained OculusNet for CNV, DME, Drusen, and Normal classes.

**Figure 13:**
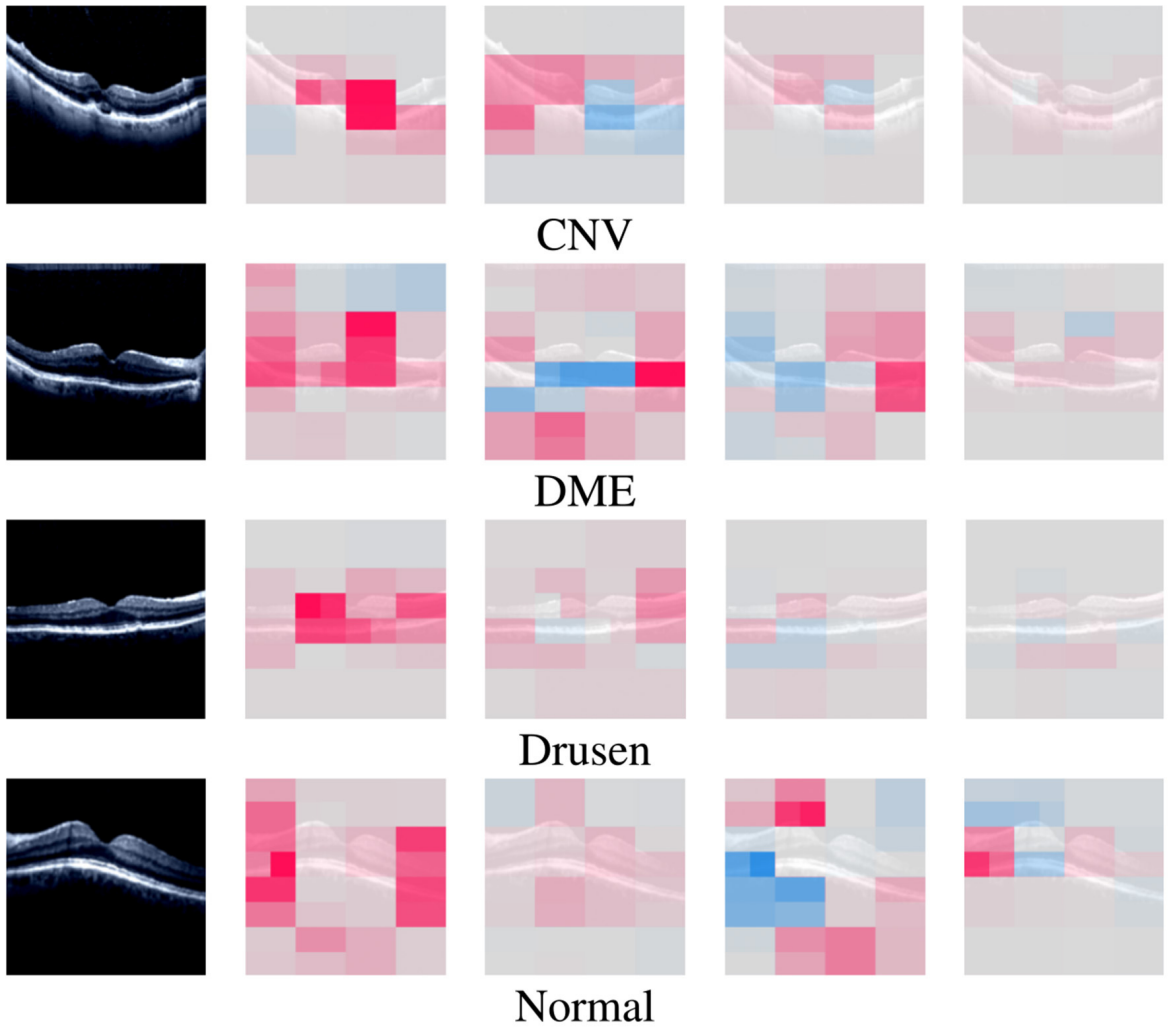
SHAP heatmaps generated by the trained OculusNet for CNV, DME, Drusen, and normal classes.

By utilizing Equation 8, the strongest pixel values are calculated from the given input image and then used to display the saliency map. Moreover, the input images used to generate the saliency map are first rescaled, and their pixel values are normalized to a range between 0 and 1 because our model was trained on rescaled and preprocessed images. The saliency map of each class on the OculusNet model is shown in Figure 12. Furthermore, for a fair comparison, Grad-CAM++ and LIME results have also been presented for each class in Figure 12. From the comparison, it can be observed that the saliency map and Grad-CAM++ show that the model was more focused on the retina layers, unlike LIME. Moreover, it is also a limitation of this study that LIME and Grad-CAM have not been optimized further; thus, as a future direction, these techniques will be explored. Additionally, SHAP analysis for each class is shown in Figure 13. From these heatmaps, it can be observed that the model is trying to focus on the retina layers for decision-making.

### 5.3 Ablation study

In this section, an ablation study was conducted to understand the impact of various architectural components on the performance of OculusNet. An ablation study systematically removes parts of the network to evaluate their contribution to the model's final performance. This approach helps identify the most critical components of the network that significantly affect its accuracy and efficiency. Three different models of OculusNet were evaluated on the test dataset, each with varying configurations and complexities. These models were designed to investigate the influence of specific layers and parameters on the network's ability to process and analyze data. The configurations of these models are detailed in Table 11, which outlines the layers and parameters involved in each model.

**Table 11:** Models architecture utilized for the ablation study.

**Layers**	**Model 1**	**Parameters**	**Model 2**	**Parameters**	**Model 3**	**Parameters**
separable_conv2d	✓	155	✓	155	✓	155
separable_conv2d_1	✓	1,344	✓	1,344	✓	1,344
separable_conv2d_2	✓	1,344	✓	1,344	✓	1,344
Max_Pooling2d	✓	0	✓	0	✓	0
Batch_Normalization	✓	128	✓	128	✓	128
separable_conv2d_3	✓	2,400	✓	2,400	-	-
separable_conv2d_4	✓	4,763	✓	4,763	-	-
Max_Pooling2d_1	✓	0	✓	0	-	-
Batch_Normalization_1	✓	256	✓	256	-	-
separable_conv2d_5	✓	8,896	-	-	-	-
separable_conv2d_6	✓	17,664	-	-	-	-
Max_Pooling2d_2	✓	0	-	-	-	-
Batch_Normalization_2	✓	512	-	-	-	-
separable_conv2d_7	-	-	-	-	-	-
separable_conv2d_8	-	-	-	-	-	-
Max_Pooling2d_3	-	-	-	-	-	-
Batch_Normalization_3	-	-	-	-	-	-
Flatten	✓	0	✓	0	✓	0
Dense	✓	9,437,312	✓	22,151,296	✓	48,664,704
Dense_1	✓	8,256	✓	8,256	✓	8,256
Dense_2	✓	260	✓	260	✓	260

Following the detailed layer and parameter configurations, Table 12 provides a summary of the total parameters, accuracy, and model size in MB for each of the three models. The ablation study emphasizes the significant impact of layer configuration and parameter count on the computational efficiency and model size of OculusNet. By comparing Model 1, Model 2, and Model 3, it becomes clear that increasing the complexity and the number of parameters greatly enlarges the model size, with Model 3 having the largest size of 185.68 MB. Conversely, Model 1, which has the fewest parameters, demonstrates a balance between model size and complexity, suggesting a more efficient architecture for applications where computational resources are limited.

**Table 12:** Obtained confusion metrics for the three models.

**Model No**.	**Accuracy (%)**	**Total parameters**	**Model size (MB)**
Model 1	83.55	9,483,263	36.18
Model 2	80.89	22,170,175	84.57
Model 3	75.24	48,676,191	185.68

**Figure 14:**
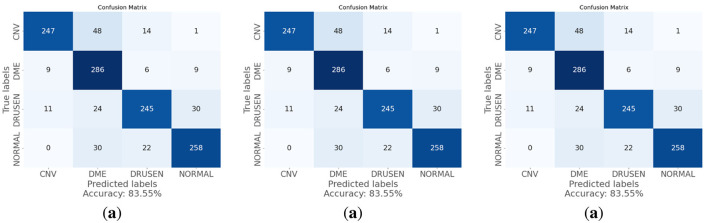
Confusion matrices of the tested models in the ablation study.

As shown in Table 12, Model 1 demonstrates the highest accuracy at 83.55% among the three models. With an accuracy of 80.89%, Model 2 exhibits a slight decrease in performance compared to Model 1. This model has improved identification for the CNV category but shows a reduction in accuracy for DME and Drusen conditions. Model 3, with an accuracy of 75.24%, reflects a decline in classification performance. The confusion matrix reveals a significant challenge in distinguishing between all conditions. Compared to Model 1, Model 2, and Model 3, the proposed architecture of the OculusNet model, which contains all layers, achieved the best testing accuracy of 95.48%. The respective confusion metrics for all three models are shown in Figure 14.

Additioanlly, Table 13 presents a comparison with recent deep-learning approaches for retinal “disease classification.” In Shin et al. (26), a semi-automated pipeline was applied to a pig-eye dataset, achieving an accuracy of 83.89%. In contrast, (29) fine-tuned several pre-trained networks on OCT B-scan images, with Xception achieving the best test accuracy of 92.00%. Moreover, Bhandari et al. (35) proposed a lightweight CNN with only 983,716 trainable parameters, achieving a test accuracy of 94.29% for classifying CNV, DME, and Drusen. By comparison, the proposed OculusNet achieves a superior accuracy of 95.48%.

**Table 13:** Comparison with other studies.

**Reference**	**Dataset**	**Approach**	**Result**
Shin et al. (26)	Pig eye dataset	Semi-automated	83.89%
Bhandari et al. (35)	OCT dataset	Lightweight CNN model	94.29%
Kang et al. (29)	OCT B- scan images	Pre-trained models	92.00%
This study	OCT	OculusNet	95.48%

### 5.4 Web deployment

**Figure 15:**
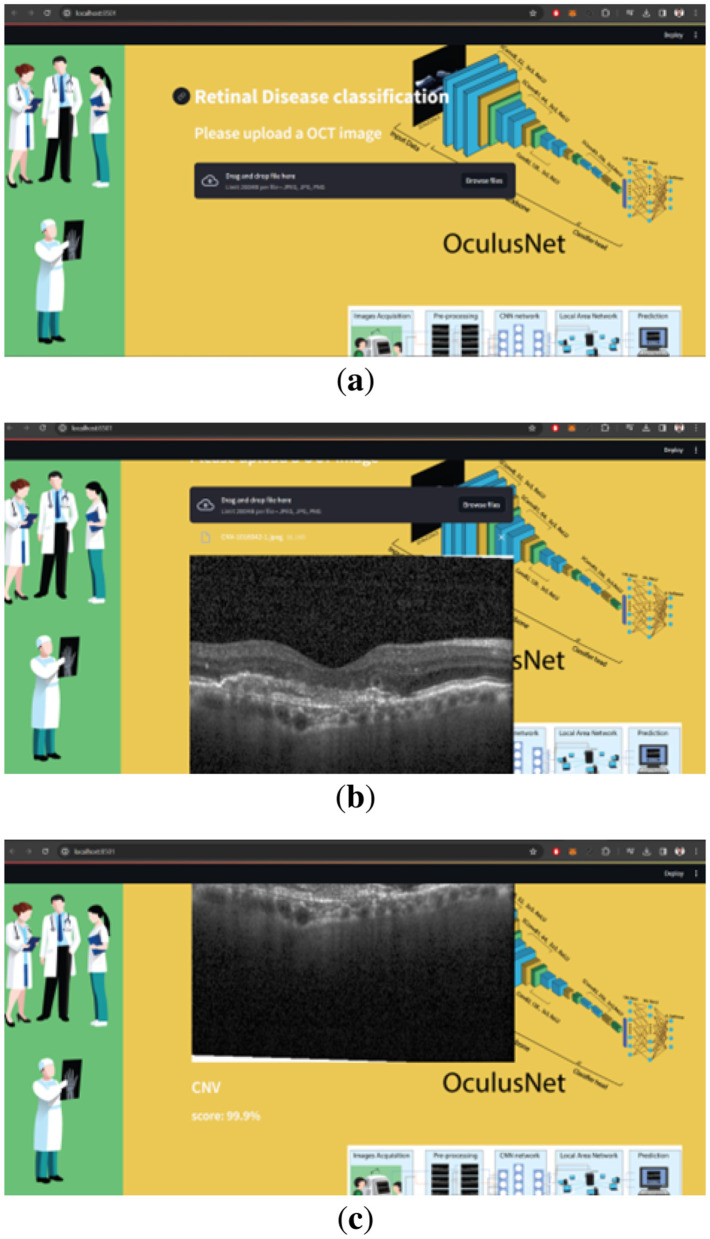
Steps for using Web page for OCT image classification. **(a)** Step 1: upload image. **(b)** Step 2: display uploaded image. **(c)** Step 3: prediction.

The web deployment of OculusNet was hosted by Streamlit, a platform known for its ease of use and efficiency in deploying data applications. The Streamlit library was employed to build an interactive web application that enables users to upload OCT images and receive classification results based on the pre-trained OculusNet model. As shown in Figure 15, the steps that will be followed to classify OCT images are outlined. Pre-trained weights from the OculusNet model are loaded into the system, ensuring that the web application utilizes the refined and optimized weights derived from extensive training sessions. A background image is set for the web application to enhance the user experience. The title and header are defined to prompt users to upload an OCT image for classification. A file uploader widget is provided for users to upload OCT images in “jpeg,” “jpg,” or “png” formats. Upon uploading, the image is displayed on the web interface. The uploaded image is passed to the classify function, which preprocesses the image and utilizes the model to predict the class of retinal disease. The classification result, along with the confidence score, is presented to the user. The confidence score is formatted to display a percentage, aiding in the interpretability of the result. Upon accessing the Streamlit web application, users encounter a clear and straightforward interface. The process is designed to be intuitive, allowing the user to easily upload an OCT image and wait for the model to classify the retinal condition. The application promptly displays the classification along with a confidence score, providing a valuable tool for preliminary diagnosis or a second opinion in clinical settings.

## 6 Conclusion

This study presented an interpretable and web-deployable approach to retinal disease classification, promising to enhance diagnostic capabilities in clinical settings. The use of saliency map visualization as an explainable AI technique improved the interpretation of the decision-making process of the proposed model. This transparency is crucial for clinical adoption, as it fosters trust and understanding among healthcare professionals. Furthermore, an ablation study was conducted on OculusNet to validate the effectiveness and robustness of the chosen architecture. To ensure a fair comparison, transfer learning was applied to four pre-trained models. The results demonstrated the superior performance of the proposed model compared to the pre-trained model, with a test accuracy of 95.48% and a validation accuracy of 98.59%. The model's performance was also evaluated using the Kappa statistic and MCC, both of which confirmed the high reliability and consistency of our model's predictions. For practical deployment, the Streamlit server was utilized to create a user-friendly interface that allows users to upload retinal OCT images and receive instant classification results. This web application has significant potential for integration into ophthalmic departments, providing an accessible and efficient tool for diagnosing retinal diseases.

## Data Availability

The datasets analyzed and utilized for this study can be found at DOI: 10.17632/rscbjbr9sj.3. Further inquiries can be directed to the corresponding authors.

## References

[1] Lee WJ. Vitamin C in Human Health and Disease: Effects, Mechanisms of Action, and New Guidance on Intake. Dordrecht: Springer Netherlands (2019). 10.1007/978-94-024-1713-5

[2] Subramanian B Devishamani C Raman R Ratra D. Association of OCT biomarkers and visual impairment in patients with diabetic macular oedema with vitreomacular adhesion. PLoS ONE. (2023) 18:1–11. 10.1371/journal.pone.0288879 37463157 PMC10353796

[3] Le Boite H Couturier A Tadayoni R Lamard M Quellec G. VMseg: Using spatial variance to automatically segment retinal non-perfusion on OCT-angiography. PLoS ONE. (2024) 19:1–11. 10.1371/journal.pone.0306794 39110715 PMC11305542

[4] Kermany DS Goldbaum M Cai W Valentim CCS Liang H Baxter SL. Identifying medical diagnoses and treatable diseases by image-based deep learning. Cell. (2018) 172:1122–1131.e9. 10.1016/j.cell.2018.02.010 29474911

[5] Farsiu S Chiu SJ O'Connell RV Folgar FA Yuan E Izatt JA. Quantitative classification of eyes with and without intermediate age-related macular degeneration using optical coherence tomography. Ophthalmology. (2014) 121:162–72. 10.1016/j.ophtha.2013.07.013 23993787 PMC3901571

[6] Xu X Li X Tang Q Zhang Y Zhang L Zhang M. Exploring laser-induced acute and chronic retinal vein occlusion mouse models: Development, temporal in vivo imaging, and application perspectives. PLoS ONE. (2024) 19:1–23. 10.1371/journal.pone.0305741 38885229 PMC11182531

[7] Alqudah A Alqudah AM AlTantawi M. Artificial Intelligence Hybrid System for Enhancing Retinal Diseases Classification Using Automated Deep Features Extracted from OCT Images. Int J Intell Syst Applic Eng. (2021) 9:91–100. 10.18201/ijisae.2021.236

[8] KanagasingamYBhuiyanAAbràmoffMDSmithRTGoldschmidtLWongTY. Progress on retinal image analysis for age related macular degeneration. Prog Retin Eye Res. (2014) 38:20–42. 10.1016/j.preteyeres.2013.10.00224211245

[9] Jayaraman V Burgner CB Carter J Borova I Bramham N Lindblad C. Widely tunable electrically pumped 1050nm MEMS-VCSELs for optical coherence tomography. In:Graham LA Lei C, editors. Vertical-Cavity Surface-Emitting Lasers XXIV. International Society for Optics and Photonics, SPIE (2020). p. 113000S. 10.1117/12.2543819

[10] Amin J Sharif M Rehman A Raza M Mufti MR. Diabetic retinopathy detection and classification using hybrid feature set. Microsc Res Tech. (2018) 81:990–6. 10.1002/jemt.23063 30447130

[11] Alqudah AM AOCT-NET. a convolutional network automated classification of multiclass retinal diseases using spectral-domain optical coherence tomography images. Med Biol Eng Comput. (2020) 58:41–53. 10.1007/s11517-019-02066-y 31728935

[12] Drexler W Fujimoto JG. State-of-the-art retinal optical coherence tomography. Prog Retin Eye Res. (2008) 27:45–88. 10.1016/j.preteyeres.2007.07.005 18036865

[13] Puliafito CA Hee MR Lin CP Reichel E Schuman JS Duker JS. Imaging of macular diseases with optical coherence tomography. Ophthalmology. (1995) 102:217–29. 10.1016/S0161-6420(95)31032-9 7862410

[14] Huang D Swanson EA Lin CP Schuman JS Stinson WG Chang W. Optical coherence tomography. Science. (1991) 254:1178–81. 10.1126/science.1957169 1957169 PMC4638169

[15] Leitgeb R Hitzenberger CK Fercher AF. Performance of Fourier domain vs. time domain optical coherence tomography. Optics Expr. (2003) 11:889–94. 10.1364/OE.11.000889 19461802

[16] Golubovic B Bouma BE Tearney GJ Fujimoto JG. Optical frequency-domain reflectometry using rapid wavelength tuning of a Cr4+:forsterite laser. Opt Lett. (1997) 22:1704–6. 10.1364/OL.22.001704 18188341

[17] Monemian M Irajpour M Rabbani H A. review on texture-based methods for anomaly detection in retinal optical coherence tomography images. Optik. (2023) 288:171165. 10.1016/j.ijleo.2023.171165

[18] Ejaz S Baig R Ashraf Z Alnfiai MM Alnahari MM Alotaibi RM. deep learning framework for the early detection of multi-retinal diseases. PLoS ONE. (2024) 19:1–23. 10.1371/journal.pone.0307317 39052616 PMC11271906

[19] Patnaik S Subasi A. Chapter 12 - Artificial intelligence-based retinal disease classification using optical coherence tomography images. In: Applications of Artificial Intelligence in Medical Imaging. Academic Press (2023). p. 305–319. 10.1016/B978-0-443-18450-5.00009-8

[20] Khalaf NB Aljobouri HK Najim MS Çankaya I. Simplified convolutional neural network model for automatic classification of retinal diseases from optical coherence tomography images. Al-Nahrain J Eng Sci. (2024) 26:314–9. 10.29194/NJES.26040314

[21] Duan J Tench C Gottlob I Proudlock F Bai L. New variational image decomposition model for simultaneously denoising and segmenting optical coherence tomography images. Phys Med Biol. (2015) 60:8901. 10.1088/0031-9155/60/22/8901 26553577

[22] Asif S Amjad K Qurrat ul A. Deep residual network for diagnosis of retinal diseases using optical coherence tomography images. Interdisc Sci. (2022) 14:906–16. 10.1007/s12539-022-00533-z 35767116

[23] Fabritius T Makita S Myllylä R Yasuno Y. Automated retinal pigment epithelium identification from optical coherence tomography images. In: Proceedings Volume 7168, Optical Coherence Tomography and Coherence Domain Optical Methods in Biomedicine XIII; SPIE BiOS. San Jose, California: SPIE (2009). 10.1117/12.808543

[24] Muni Nagamani G Rayachoti E. Deep learning network (DL-Net) based classification and segmentation of multi-class retinal diseases using OCT scans. Biomed Signal Process Control. (2024) 88:105619. 10.1016/j.bspc.2023.105619

[25] Wang M Zhu W Yu K Chen Z Shi F Zhou Y. Semi-supervised capsule cGAN for speckle noise reduction in retinal OCT images. IEEE Trans Med Imag. (2021) 40:1168–83. 10.1109/TMI.2020.3048975 33395391

[26] Shin C Gerber MJ Lee YH Rodriguez M Pedram SA Hubschman JP. Semi-automated extraction of lens fragments via a surgical robot using semantic segmentation of OCT images with deep learning - experimental results in ex vivo animal model. IEEE Robot Autom Lett. (2021) 6:5261–8. 10.1109/LRA.2021.3072574 34621980 PMC8492005

[27] Adel A Soliman MM Khalifa NEM Mostafa K. Automatic classification of retinal eye diseases from optical coherence tomography using transfer learning. In: 2020 16th International Computer Engineering Conference (ICENCO). IEEE (2020). p. 37–42. 10.1109/ICENCO49778.2020.9357324

[28] Islam KT Wijewickrema S O'Leary S. Identifying diabetic retinopathy from OCT images using deep transfer learning with artificial neural networks. In: 2019 IEEE 32nd International Symposium on Computer-Based Medical Systems (CBMS). IEEE (2019). p. 281–286. 10.1109/CBMS.2019.00066

[29] Kang NY Ra H Lee K Lee JH Lee WK Baek J. Classification of pachychoroid on optical coherence tomography using deep learning. Graefe's Arch Clin Exper Ophthalmol. (2021) 259:1803–9. 10.1007/s00417-021-05104-4 33616757

[30] Wu Q Zhang B Hu Y Liu B Cao D Yang D. Detection of morphologic patterns of diabetic macular edema using a deep learning approach based on optical coherence tomography images. Retina. (2021) 41:1110–7. 10.1097/IAE.0000000000002992 33031250 PMC8078116

[31] Hassan SAE Akbar S Gull S Rehman A Alaska H. Deep learning-based automatic detection of central serous retinopathy using optical coherence tomographic images. in 2021 1st International Conference on Artificial Intelligence and Data Analytics (CAIDA). IEEE (2021). 206–211.

[32] Jawad MA Khursheed F Nawaz S Mir AH. Towards improved fundus disease detection using Swin Transformers. Multimed Tools Appl. (2024) 83:78125–59. 10.1007/s11042-024-18627-9

[33] Abdul Jawad M Khursheed F. Deep and dense convolutional neural network for multi category classification of magnification specific and magnification independent breast cancer histopathological images. Biomed Signal Process Control. (2022) 78:103935. 10.1016/j.bspc.2022.103935

[34] Abdul Jawad M Khursheed F. A novel approach for color-balanced reference image selection for breast histology image normalization. Biomed Signal Process Control. (2024) 94:106299. 10.1016/j.bspc.2024.106299

[35] Bhandari M Shahi TB Neupane A. Evaluating retinal disease diagnosis with an interpretable lightweight CNN model resistant to adversarial attacks. J Imaging. (2023) 9:219. 10.3390/jimaging9100219 37888326 PMC10607865

[36] Kermany D. Labeled optical coherence tomography (OCT) and chest X-ray images for classification. Mendeley data (2018).

[37] Le DN Parvathy VS Gupta D Khanna A Rodrigues JJPC Shankar K. IoT enabled depthwise separable convolution neural network with deep support vector machine for COVID-19 diagnosis and classification. Int J Mach Learn Cybern. (2021) 12:3235–48. 10.1007/s13042-020-01248-7 33727984 PMC7778504

[38] Li Q Ning J Yuan J Xiao L. A depthwise separable dense convolutional network with convolution block attention module for COVID-19 diagnosis on CT scans. Comput Biol Med. (2021) 137:104837. 10.1016/j.compbiomed.2021.104837 34530335 PMC8425669

[39] Umair M Khan MS Ahmed F Baothman F Alqahtani F Alian M. Detection of COVID-19 using transfer learning and grad-CAM visualization on indigenously collected x-ray dataset. Sensors. (2021) 21:5813. 10.3390/s21175813 34502702 PMC8434081

[40] Yang J Wang G Xiao X Bao M Tian G. Explainable ensemble learning method for OCT detection with transfer learning. PLoS ONE. (2024) 19:1–17. 10.1371/journal.pone.0296175 38517913 PMC10959366

[41] Bhandari M Shahi TB Siku B Neupane A. Explanatory classification of CXR images into COVID-19, Pneumonia and Tuberculosis using deep learning and XAI. Comput Biol Med. (2022) 150:106156. 10.1016/j.compbiomed.2022.106156 36228463 PMC9549800

